# Antiplatelet and Antithrombotic Activities of Non-Steroidal Anti-Inflammatory Drugs Containing an *N*-Acyl Hydrazone Subunit

**DOI:** 10.3390/molecules19022089

**Published:** 2014-02-17

**Authors:** Rafael Consolin Chelucci, Luiz Antônio Dutra, Maria Elisa Lopes Pires, Thais Regina Ferreira de Melo, Priscila Longhin Bosquesi, Man Chin Chung, Jean Leandro dos Santos

**Affiliations:** Departamento de Fármacos e Medicamentos, Faculdade de Ciências Farmacêuticas–UNESP, Rodovia Araraquara Jaú Km, Araraquara, SP, 01, 14801-902, Brazil; E-Mails: rafaelchelucci@hotmail.com (R.C.C.); luizdutra_qf@yahoo.com.br (L.A.D.); mariaelisalopes@yahoo.com.br (M.E.L.P.); trfmelo@gmail.com (T.R.F.M.); bosquesi@fcfar.unesp.br (P.L.B.); chungmc@fcfar.unesp.br (M.C.C.)

**Keywords:** hydrazone, antiplatelet, antithrombotic, NSAIDs, bleeding time

## Abstract

Nonsteroidal anti-inflammatory drugs (NSAIDs) **1**–**5** containing an N-acyl hydrazone subunit were prepared and their antiplatelet and antithrombotic activities assessed *in vitro* and *in vivo*. Compounds **1**–**5** inhibited the platelet aggregation induced by adenosine diphosphate and/or arachidonic acid, with inhibition rates of 18.0%–61.1% and 65.9%–87.3%, respectively. Compounds **1** and **5** were the most active compounds, inhibiting adenosine-diphosphate-induced platelet aggregation by 57.2% and 61.1%, respectively. The inhibitory rates for arachidonic-acid-induced platelet aggregation were similar for compound **2** (80.8%) and acetylsalicylic acid (ASA, 80%). After their oral administration to mice, compounds **1**, **3**, and **5** showed shorter mean bleeding times than ASA. Compounds **1** and **5** also protected against thromboembolic events, with survival rates of 40% and 33%, respectively, compared with 30% for ASA. In conclusion, these results indicate that these novel NSAIDs containing an NAH subunit may offer better antiplatelet and antithrombotic activities than ASA.

## 1. Introduction

**Figure 1:**
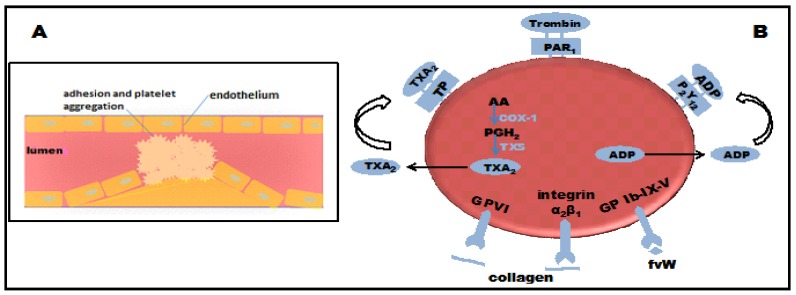
(**A**) Platelets are activated and start to adhere to the endothelial wall in response to vessel injury; and (**B**) platelet aggregation pathways.

Cardiovascular disease and stroke are major causes of morbidity and mortality in high-income countries [1,2]. Although many factors contribute to the development of cardiovascular disease, thrombus formation is the main trigger event in acute coronary syndrome and stroke. Injury to the endothelial wall causes platelets to activate and adhere to the exposed endothelium, leading to thrombus formation [3,4] (Figure 1). Platelet aggregation plays an important role in the pathogenesis of thromboembolic disorders [5]. Several endogenous compounds, including thrombin, thromboxane A_2,_ collagen, von Willebrand factor, and ADP stimulate platelet aggregation through different pathways (Figure 1). Under normal physiological conditions, platelets maintain a balance between anti-aggregatory and pro-aggregatory activities. Nonsteroidal anti-inflammatory drugs (NSAIDs) inhibit the thromboxane A_2_ pathway and therefore inhibit platelet aggregation. However, NSAIDs also reduce the expression of prostaglandin I_2_, an anti-aggregatory mediator, in the endothelial wall [6,7].

The long-term administration of NSAIDs, except for aspirin (acetylsalicylic acid [ASA]), a covalent cyclooxygenase 1 (COX-1) inhibitor that is used for primary and secondary cardiovascular prophylaxis [8], has deleterious effects on the cardiovascular system. Rofecoxib, a COX-2 inhibitor, was withdrawn from the market in 2004 because several controlled clinical trials showed that it was associated with an increased risk of cardiovascular events [9]. Consequently, the cardiovascular safety of COX-2 inhibitors and classical NSAIDs has been vigorously debated by the scientific community [10]. Several clinical studies and meta-analyses have since shown that NSAIDs are associated with a greater risk of cerebrovascular and cardiovascular events compared with placebos [11,12,13]. Furthermore, the long-term administration of some NSAIDs, including ibuprofen and diclofenac, is associated with an increased risk of stroke and cardiovascular-related death [11]. Ibuprofen and COX-2 inhibitors (e.g., celecoxib, rofecoxib, and lumiracoxib) have also been associated with myocardial infarction. An increased risk of cardiovascular events was also described for ketoprofen [14]. Although the data is controversial, naproxen was reported to be the least harmful NSAID in several clinical studies [13,15]. Despite these deleterious effects, NSAIDs are still frequently prescribed for the treatment of osteoarthritis and other chronic inflammatory diseases, although they may be contraindicated in older patients (>75 years) and in patients with hypertension, chronic renal disease, prior myocardial infarction, cerebrovascular disease, rheumatoid arthritis, or gastrointestinal ulcers [16,17].

**Figure 2:**
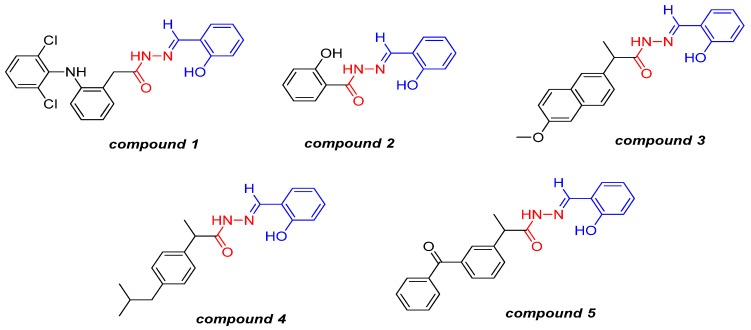
Structures of compounds **1**–**5**, which were obtained by molecular hybridization of a NSAID, ASA, and an NAH prototype.

We previously described the synthesis and anti-inflammatory and analgesic activities of novel NSAIDs containing an *N*-acyl hydrazone (NAH) subunit (Figure 2). The analgesic activities of these compounds was evaluated in terms of their inhibition of acetic-acid-induced abdominal constriction in mice. After oral administration at a dose of 100 μmol/kg, all the compounds displayed superior analgesic activity to that of their parent NSAID. We also evaluated compound **3** using the rat carrageenan-induced paw edema model, and found that it reduced the extent of inflammation by 35.9% and 52.8% at 2 and 4 h, respectively (unpublished data).

The antiplatelet activity of NSAID derivatives containing an NAH subunit has been extensively described in the literature. An “ideal antiplatelet” drug must be able to block platelet thrombogenesis by different pathways to avoid “platelet resistance”. Moreover, the drug must not interfere with hemostasis and/or wound healing. In this paper, we describe the antiplatelet and antithrombotic activities of compounds **1**–**5**, which were designed as novel anti-inflammatory and analgesic compounds without gastrotoxic effects. Compounds **1**–**5** were designed using a molecular hybridization approach, in which three different pharmacophores, represented by their parent NSAID, ASA, and NAH structures, were combined into a single structure. We used ASA as one of the components because of its established use in primary and secondary cardiovascular prophylaxis. The discovery of NSAIDs with cardioprotective properties, which do not prolong bleeding time, is a challenge in the field of medicinal chemistry, and may represent a new approach to the long-term treatment of several inflammatory diseases.

## 2. Results and Discussion

### 2.1. Antiplatelet Activity

The antiplatelet activity of each compound was evaluated using rat platelet-rich plasma and aggregation was induced with ADP (10 μM) or arachidonic acid (AA; 100 μM) [18,19]. In this *in vitro* assay, compounds **1**–**5** (and ASA as the control) were tested at doses of 150 μM. Although we initially prepared dose–response curves for each compound at doses of 50–250 μM, only doses of ≥ 150 μM inhibited platelet aggregation, and the levels of platelet inhibition were similar at doses of 150, 200, and 250 μM. Therefore, the effects of compounds **1**–**5** were evaluated *in vitro* at 150 μM. As shown in Table 1, all the compounds inhibited the platelet aggregation induced by ADP or AA, with inhibition rates of 18.0%–61.1% and 65.9%–87.3% for ADP- and AA-induced platelet aggregation, respectively. Compounds **1** and **5** were the strongest inhibitors of ADP-induced platelet aggregation, with rates of 57.2% and 61.1%, respectively. The inhibition of AA-induced platelet aggregation was similar for compound **2** (80.8%) and ASA (80%). Clopidogrel did not inhibit platelet aggregation in this assay because it is a prodrug (data not shown).

**Table 1:** Effects of NAH derivatives **1**–**5** (150 μM) and ASA (150 μM) on the platelet inhibition induced by ADP (10 μM) or AA (100 μM) in platelet-rich plasma.

Compound	n	Concentration (μM)	ADP-Induced Platelet Aggregation (% Inhibition)	AA-Induced Platelet Aggregation (% Inhibition)
Control	3	–	0	0
ASA	3	150	0	80 ± 1.6 ^*^
**1**	3	150	57.2 ± 2.1 ^*^^,^^†^	71.4 ± 2.2 ^*^
**2**	3	150	29.5 ± 1.8 ^*^^,^^†^	80.8 ± 1.1 ^*^
**3**	3	150	30.5 ± 2.3 ^*^^,^^†^	66.8 ± 1.7 ^*^
**4**	3	150	18.0 ± 1.7 ^*^^,^^†^	67.6 ± 2.1 ^*^
**5**	3	150	61.1 ± 2.5 ^*^^,^^†^	65.9 ± 1.3 ^*^

Data are expressed as the means ± standard errors of the means. Statistical differences between the experimental and control groups were evaluated by analysis of variance followed by the Tukey test. ^*^ *p* < 0.01 *vs.* the control group; ^†^ *p* < 0.01 *vs.* ASA. NAH, *N*-acylhydrazone; ASA, acetylsalicylic acid; ADP, adenosine diphosphate; AA, arachidonic acid.

The antiplatelet activities of NAH have been described in previous studies [20]. Several studies have shown that the presence of this subunit enhances antiplatelet activity [21,22,23]. The NAH subunit is thought to inhibit antiplatelet activity because of its high affinity for COX-1 and greater inhibition of TXA_2_ formation than other COX-1 inhibitors [24]. It is also believed that the NAH subunit is capable of acting as a calcium chelator by decreasing intracellular calcium concentration and interfering with the process of platelet activation and aggregation [25]. Platelet aggregation plays an important role in the pathogenesis of thromboembolic disorders. Several reports have demonstrated that some NAH derivatives can inhibit the platelet aggregation induced by ADP or AA [26,27,28,29]. The binding of ADP to the purinergic P2Y_12_ receptor on platelets activates adenylyl cyclase and the production of cAMP, culminating in platelet aggregation. However, prostaglandin I_2_ interacts with prostaglandin receptors to suppress cAMP production, thus inhibiting platelet aggregation [30]. Clopidogrel, a P2Y_12_ receptor antagonist, is one of the most widely prescribed platelet inhibitors. Although it has beneficial benefits in preventing platelet aggregation, several adverse effects, including bleeding, neutropenia, and thrombotic thrombocytopenic purpura, may limit its use as an antiplatelet therapy [31]. “Resistance” to clopidogrel is another problem that may complicate clopidogrel therapy [32].

### 2.2. Bleeding Time in Mice

The bleeding time in anesthetized mice was measured after a small incision was made in the tail with a scalpel blade. The time taken from the tail tip incision to the cessation of bleeding was recorded as the total bleeding time. In this experiment, compounds **1**–**5** and ASA were orally administered at doses of 100 μmol/kg bodyweight. Although dose–response curves were constructed for each compound at doses of 10–300 μmol/kg, only doses of ≥ 100 μmol/kg markedly influenced the bleeding time, without unacceptably shortening or prolonging the bleeding time. As shown in Table 2, all the compounds increased bleeding time relative to the negative control group. The bleeding times for compounds **1**, **3**, and **5** were similar, and were shorter than that of ASA, the positive control. Of these compounds, compounds **2** and **4** had the greatest effect on prolonging bleeding time.

**Table 2:** Effects of compounds **1**–**5** and ASA on bleeding time *in vivo*.

Compounds	Bleeding Time (s)
**Negative control**	343 ± 11.5
**ASA**	818 ± 21.8 ^*^
**1**	642 ± 18.1 ^*,†^
**2**	1271 ± 24.9 ^*^
**3**	671 ± 16.7 ^*,†^
**4**	1178 ± 25.3 ^*^
**5**	653 ± 14.1 ^*,†^

Data are expressed as means ± standard errors of the means. Statistical differences between the experimental and control groups were evaluated by analysis of variance with Dunnett’s test. * *p* < 0.01 *vs.* the negative control group. ^†^ *p* < 0.01 *vs.* ASA. ASA, acetyl-salicylic acid.

The main challenge for antiplatelet and antithrombotic therapy is to develop new drugs capable of preventing thromboembolic events without increasing bleeding. Dual antiplatelet therapies, typically ASA and clopidogrel, are necessary for some cardiovascular diseases, including acute coronary syndrome, although this strategy increases the risk of bleeding [33,34]. Many patients are also prescribed more than two drugs, including NSAIDs, increasing the risk of severe adverse events [35]. NSAIDs such as celecoxib, ibuprofen, flufenamic acid, naproxen, nimesulide, and piroxicam may interfere with the antiplatelet activity of ASA in terms of its inhibition of COX-1, thus increasing the risk of atherothrombotic events [36]. Therefore, a NSAID that offers anti-inflammatory/analgesic activities in combination with antiplatelet effects is potentially useful for the treatment of chronic inflammatory conditions.

Because compounds **1**, **3**, and **5** had shorter bleeding times than ASA *in vivo* and inhibited platelet aggregation *in vitro*, they were subjected to additional *in vivo* studies to characterize their antithrombotic effects.

### 2.3. Collagen- and Epinephrine-Induced Pulmonary Thromboembolism Model in Mice

The antithrombotic effects of the test compounds were evaluated using a pulmonary thromboembolism model, which was induced by injecting mice with a mixture of collagen (11 mg/kg bodyweight) and epinephrine (0.7 mg/kg bodyweight). These doses of collagen and epinephrine were chosen based on those used in previous studies [37,38]. The mixture was rapidly injected into the tail vein, causing death or paralysis without a righting reflex because pulmonary thrombi were formed. The test compounds and ASA were orally administered at doses of 100 µmol/kg bodyweight (0.1 mL/10 g). Although dose–response curves were constructed for each compound at doses of 50–300 μmol/kg bodyweight, only doses of ≥ 100 μmol/kg bodyweight protected the mice against thromboembolic events, and the reductions in thrombotic events were similar at doses of 200 and 300 μmol/kg bodyweight. Therefore, the antithrombotic effects of compounds **1**, **3**, and **5**, and ASA were evaluated after an oral dose of 100 μmol/kg bodyweight. As shown in Table 3, ASA and compounds **1** and **5** protected the mice against thromboembolic events in this model. The survival rates, which were calculated as the percentage of surviving mice without paralysis, was 30%, 40%, and 33%, in mice treated with ASA, compound **1**, and compound **5**, respectively. Compound **3** did not protect the mice against pulmonary thromboembolism in this model at any of the concentrations tested.

**Table 3:** Antithrombotic effects of ASA and compounds **1**, **3**, and **5** on pulmonary thromboembolism induced by a mixture of collagen and epinephrine in mice *in vivo.*

Compound	Survival Rate (%) ^*^
Control	20 ± 1
ASA	30 ± 3 ^†^
**1**	40 ± 1 ^†,‡^
**3**	20 ± 2
**5**	33 ± 2 ^†^

Data are expressed as means ± standard errors of the means. Statistical differences between the experimental and control groups were evaluated by analysis of variance and Dunnett’s test. * The percentage of mice surviving without paralysis, where the loss of the righting reflex was considered as an indication of paralysis. ^†^ *p* < 0.05 *vs.* the control group; ^‡^ *p* < 0.05 *vs.* ASA.

These results indicate that compounds **1** and **5** inhibited thrombosis, and their inhibitory effects were related to the inhibition of platelet aggregation.

## 3. Experimental

### 3.1. General Information

#### 3.1.1. Reagents and equipaments

ADP, arabic gum, arachidonic acid, acetylsalicylic acid, collagen, dimethylsulfoxide, ephinephrine, ethanol, halothane, trisodium citrate, saline. All reagents and solvents were bought in Sigma-Aldrich (St. Louis , MO, USA).

Platelet aggregation was measured using Chrono-Log aggregometer model 490 (Harvertown, PA, USA). The platelet-rich plasma (PRP) was prepared by centrifugation using Fanem Excelsa Baby I model 206BL (São Paulo, Brazil).

### 3.2. Preparation of Compounds **1**–**5**

The *N*-acylhydrazone derivatives **1**–**5** were prepared by condensation between hydrazides and salicylaldehydes in excellent yields (61%–82%) and characterized by appropriate analytical methods such as hydrogen nuclear magnetic resonance (^1^H-NMR), infrared (IR), mass spectrometry (MS) and elemental analysis. In this study, all compounds were also analyzed by high-performance liquid chromatography (HPLC) and their purity was confirmed to be over 98.5% [39].

### 3.3. Animals

Adult male Wistar rats (150–200 g) and Swiss male albino mice (20–35 g) were used in the experiments. They were housed in cages under a 12 h light: 12 h dark cycle (lights on at 6 am) in a controlled-temperature room (22 ± 2 °C). The rats and mice had free access to food and water. Groups of five animals were used in each test group and the control animals received vehicle only. The experiments were performed after the protocol was approved by the local Institutional Ethics Committee. All experiments were performed in accordance with the current guidelines for the care of laboratory animals and the ethical guidelines for the investigation of experimental pain in conscious animals.

### 3.4. Pharmacology

#### 3.4.1. Antiplatelet Activity

Platelet aggregation was monitored at 37 °C by the turbidimetric method of Born and Cross [19] using a Chrono-Log aggregometer model 490 (Harvertown, PA, USA). Blood was collected from anesthetized rats by aorta puncture using syringes containing 3.8% trisodium citrate (9:1 v/v). Platelet-rich plasma (PRP) was prepared by centrifugation at 300 *×g* for 10 min at room temperature. The platelet-poor plasma (PPP) was prepared by centrifugation of the pellet at 1600 *×g* for 10 min at room temperature. In a cuvette, PRP (400 µL) was pre-incubated with compounds (150 μM), acetylsalicylic acid (150 μM; positive control) or vehicle DMSO (0.1% v/v) at 37 °C for 3 min with continuous stirring at 1000 rpm. Platelet aggregation was induced by ADP (10 μM) and arachidonic acid (AA) and the aggregation curve was determinated for 5 min after agonist addition. The results were expressed as mean ± SD of three independent experiments. The inhibition percentage and were calculated as follow: 

The inhibition ratio % = [(platelet aggregation of control group − platelet aggregation of treated groups)/platelelet aggregation of control group 100%] × 100. The Statistical analysis was performed with ANOVA followed by Tukey’s test.

#### 3.4.2. Bleeding Time

Bleeding time in mice was evaluated using an adapted method described by Dejana and co-workers [40]. All compounds and acetylsalycilic acid were administrated orally at 100 μmol/Kg as a suspension in a mixture of 5% (mass/vol) of arabic gum in saline. All compounds and vehicle were given orally 60 min prior experiment. The incision was made 2 mm from tip of mice tail and the blood was soaked on a filter paper, which was monitored at an interval of 10–15 s till the bleeding stopped. The time elapsed from the tail tip incision to the stoppage of bleeding was recorded as the bleeding time [40,41]. The results are the bleeding time mean ± SD expressed in s of ten animals in each group. The Statistical analysis was performed with ANOVA followed by Dunnett’s test (*p* < 0.01).

#### 3.4.3. Pulmonary Thromboembolism Model Induced by Collagen-Ephinephrine Mixture

Pulmonary thromboembolism in mice was induced according method previously described [18,37,38]. *N*–acylhydrazones derivatives (**1**–**5**) were given orally (200 µmol/20g, 0.2 mL/20 g) as a suspension in 5% arabic gum in saline (vehicle), 60 min before intravenous injection of the thrombogenic stimulus. Thrombotic event was induced by mixture of collagen (11 mg/Kg) plus ephinephrine (0.7 mg/Kg). The dose of the mixture was determinated, using curve dose/response, as responsible to reproduce 80%–90% mortality rate or animal standstill from the formation of pulmonary thrombi. The loss of the righting reflex was considered an indication of paralysis in control group. The survival rate or animal standstill was evaluated 15 min after thrombotic event. The protection against acute pulmonary thromboembolism (percentage) was calculated as follows: (1 − [paralyzed + death animals]/total animals) × 100. Results shown represent as the mean ± SD of two groups of ten animals each. *p* < 0.05. The Statistical analysis was performed with ANOVA followed by Dunnett’s test (*p* < 0.05).

## 4. Conclusions

Five NSAID derivatives **1**–**5** containing an NAH subunit were prepared and their antiplatelet and antithrombotic effects evaluated. All five compounds inhibited the platelet aggregation induced with ADP (10 μM) or AA (100 μM) *in vitro*. Compounds **1** and **5** were the most active compounds, inhibiting ADP-induced platelet aggregation by 57.2% and 61.1%, respectively. The effects of compound **2** on AA-induced platelet aggregation were similar to that of ASA. Compounds **1**, **3**, and **5** had shorter bleeding times than ASA (positive control), whereas compounds **2** and **4** prolonged the bleeding time (both > 1178 s). The antithrombotic effects of compounds **1**, **3**, and **5** were evaluated using a pulmonary thromboembolism model induced by rapidly injecting mice with a mixture of collagen (11 mg/kg bodyweight) and epinephrine (0.7 mg/kg bodyweight). Compounds **1** and **5** protected the mice against thromboembolic events, with survival rates of 40% and 33%, respectively, whereas the survival rate in mice treated with ASA was 30%. Our data suggest that the inhibition of thrombus formation is dependent on the inhibition of platelet aggregation. Based on these findings, we believe that compounds **1**, **3**, and **5** are candidate drugs with combined antiplatelet and antithrombotic activities and a shorter bleeding time than ASA.
